# Effects of Combinational Use of Additional Differential Diagnostic Generators on the Diagnostic Accuracy of the Differential Diagnosis List Developed by an Artificial Intelligence–Driven Automated History–Taking System: Pilot Cross-Sectional Study

**DOI:** 10.2196/49034

**Published:** 2023-08-02

**Authors:** Yukinori Harada, Shusaku Tomiyama, Tetsu Sakamoto, Shu Sugimoto, Ren Kawamura, Masashi Yokose, Arisa Hayashi, Taro Shimizu

**Affiliations:** 1 Department of Diagnostic and Generalist Medicine Dokkyo Medical University Mibu, Shimotsugagun Japan; 2 Department of Internal Medicine Nagano Chuo Hospital Nagano Japan

**Keywords:** collective intelligence, differential diagnosis generator, diagnostic accuracy, automated medical history taking system, artificial intelligence, AI

## Abstract

**Background:**

Low diagnostic accuracy is a major concern in automated medical history–taking systems with differential diagnosis (DDx) generators. Extending the concept of collective intelligence to the field of DDx generators such that the accuracy of judgment becomes higher when accepting an integrated diagnosis list from multiple people than when accepting a diagnosis list from a single person may be a possible solution.

**Objective:**

The purpose of this study is to assess whether the combined use of several DDx generators improves the diagnostic accuracy of DDx lists.

**Methods:**

We used medical history data and the top 10 DDx lists (index DDx lists) generated by an artificial intelligence (AI)–driven automated medical history–taking system from 103 patients with confirmed diagnoses. Two research physicians independently created the other top 10 DDx lists (second and third DDx lists) per case by imputing key information into the other 2 DDx generators based on the medical history generated by the automated medical history–taking system without reading the index lists generated by the automated medical history–taking system. We used the McNemar test to assess the improvement in diagnostic accuracy from the index DDx lists to the three types of combined DDx lists: (1) simply combining DDx lists from the index, second, and third lists; (2) creating a new top 10 DDx list using a 1/n weighting rule; and (3) creating new lists with only shared diagnoses among DDx lists from the index, second, and third lists. We treated the data generated by 2 research physicians from the same patient as independent cases. Therefore, the number of cases included in analyses in the case using 2 additional lists was 206 (103 cases × 2 physicians’ input).

**Results:**

The diagnostic accuracy of the index lists was 46% (47/103). Diagnostic accuracy was improved by simply combining the other 2 DDx lists (133/206, 65%, *P*<.001), whereas the other 2 combined DDx lists did not improve the diagnostic accuracy of the DDx lists (106/206, 52%, *P*=.05 in the collective list with the 1/n weighting rule and 29/206, 14%, *P*<.001 in the only shared diagnoses among the 3 DDx lists).

**Conclusions:**

Simply adding each of the top 10 DDx lists from additional DDx generators increased the diagnostic accuracy of the DDx list by approximately 20%, suggesting that the combinational use of DDx generators early in the diagnostic process is beneficial.

## Introduction

Diagnostic errors, defined as “the failure to (a) establish an accurate and timely explanation of the patient’s health problem(s) or (b) communicate that explanation to the patient,” are common worldwide patient safety issues in outpatients [1-8]. Since history-taking failure and hypothesis generation failure or failure to consider the correct diagnosis are the most important contributing factors to diagnostic errors [9-13], they can be major targets of intervention to reduce diagnostic errors. Indeed, previous studies reported that reminding physicians of the considerable diagnoses before they started testing hypotheses increased the number of differential diagnoses and improved diagnostic accuracy irrespective of case difficulty [14-16]. From this point of view, automated medical history–taking systems with differential diagnosis (DDx) generators, which can automatically gather important information about a patient’s medical history and suggest possible differential diagnoses, are promising information technologies for reducing diagnostic errors [2].

Automated medical history–taking systems have a long history of development, and their usefulness and quality have been validated [17,18]. Furthermore, the recent evolution of artificial intelligence (AI) using new machine learning methods has empowered the quality of these systems. Indeed, according to previous studies, automated medical history–taking systems showed better performance in taking patient histories than physicians [19,20], increased the number of questions generated by resident physicians during their interviews [21], and supported better diagnostic decisions of physicians in emergency and outpatient department settings [22,23].

However, low diagnostic accuracy is a major concern in automated medical history–taking systems with DDx generators. Several systematic reviews have consistently reported low diagnostic accuracy regarding DDx generators and symptom checkers, another type of DDx generator that generates possible diagnoses based on the patient’s input [24-26]. This is also the case with automated medical history–taking systems. In fact, a previous study reported that there were only 50% of cases in which the correct diagnosis was included in 10 DDx lists generated by the automated medical history–taking system in patients who visited the outpatient department and were unexpectedly hospitalized within 14 days after the index visit [23]. This low accuracy is problematic because the DDx list and medical history generated by the automated medical history–taking system may reduce the diagnostic accuracy of physicians in cases where the automated medical history–taking system does not include the correct diagnosis in the DDx list [27,28]. Therefore, methods to improve the accuracy of the DDx list of automated medical history–taking systems are warranted. The best method appears to be to improve algorithms using machine learning with high-quality supervised data; however, this is unlikely to be achieved in a short period of time. Therefore, other methods must be explored.

Extending the concept of collective intelligence to the field of DDx generators such that the accuracy of judgment becomes higher when accepting an integrated diagnosis list from multiple people than when accepting a diagnosis list from a single person may be a possible solution [29-33]. However, to the best of our knowledge, no study has assessed whether the collective intelligence of differential diagnostic generators works well in improving diagnostic accuracy. Therefore, we conducted this study to assess whether the combined use of different diagnostic generators can improve the diagnostic accuracy of the DDx list of an automated medical history–taking system.

## Methods

### Study Design and Participants

This pilot study used 2 differential diagnostic generators and 1 AI-driven automated medical history–taking system with a differential diagnostic generator. Data on medical histories and DDx lists (index lists) developed by the AI-driven automated medical history–taking system were retrospectively collected at Nagano Chuo Hospital. We included patients aged 18 years or older who used the AI-driven automated medical history–taking system when visiting the outpatient clinic of the hospital for new problems within the routine care setting between January 1, 2020, and December 31, 2020, and were admitted within 30 days from the initial visit. We excluded the data of patients whose final diagnoses were unknown or for whom the AI-driven automated medical history–taking system developed a DDx list that contained less than 10 differential diagnoses, indicating that automated medical history–taking was not fully conducted. We set inclusion and exclusion criteria to effectively select data suitable for this analysis.

### Ethics Approval

The study complied with the principles of the Declaration of Helsinki. The research ethics committees of Dokkyo Medical University (2022-001) and Nagano Chuo Hospital approved this study (NCR202204) and waived the requirement for written informed consent from the participants because we used an opt-out method. We informed the participants by providing them with detailed information about the study in the outpatient waiting area at Nagano Chuo Hospital and on the hospital’s website.

### Data Collection

We extracted data on age (categorized into 5 groups: 18-29, 30-39, 40-49, 50-64, and 65 years or older for anonymization), sex, medical history, and a DDx list generated by the AI-driven automated medical history–taking system. First, 2 research physicians (YH and SS) independently determined the final diagnoses by reviewing the medical records and discharge summaries of patients who fulfilled the inclusion criteria. Disagreements were resolved through discussions. When there was a disagreement between the research physicians’ diagnosis and the treating physician’s diagnosis, the research physicians’ diagnosis was deemed the final diagnosis for the purposes of this study. Second, the other research physicians (T Sakamoto and ST) independently developed 2 additional DDx lists (the second and third lists) per case using 2 DDx generators (Isabel Pro and the AI diagnostic support system for general internal medicine) based on the patient’s age, sex, and medical history generated by the AI-driven automated medical history–taking system without reading the index lists generated by the AI-driven automated medical history–taking system. Medical histories generated by the AI-driven automated medical history–taking system were written in Japanese; therefore, when using Isabel Pro, the research physicians entered keywords by translating Japanese into English themselves. The input words were selected at the discretion of the research physicians. Every top 10 DDx list generated by the 2 differential diagnostic generators was extracted and stored as a PDF file or a screenshot. Subsequently, 4 research physicians (YH, RK, MY, and AH) checked whether there was a shared DDx among the 3 lists. Conflicts were resolved through discussions. In addition, 2 research physicians (YH and ST) coded the chief concerns and final diagnoses using the International Classification of Primary Care 3rd Revision and International Classification of Diseases 11th Revision codes. Additionally, 2 independent physician researchers (YH and T Sakamoto) classified the final diagnoses into categories of common and uncommon diseases. Any discrepancies were addressed through collaborative discussion. Uncommon diseases were defined, in accordance with the European definition of a rare disease, as those affecting no more than 1 individual per 2000 people.

### Used Tools

In this study, we opted for 3 differential diagnostic generators that incorporated certain AI algorithms: the AI-driven automated medical history–taking system, Isabel Pro, and the AI diagnostic support system for general internal medicine. This selection was predicated on the feasibility of these systems, given the pilot nature of our study. The specific algorithms used within these systems were not disclosed. Despite the apparent coverage of these systems beyond internal diseases, the validation of these 3 systems primarily pertained to internal diseases, as demonstrated in studies involving actual patients or clinical vignettes [23,26,34]. The AI-driven automated medical history–taking system used in this study was developed by Ubie Inc. Details of the AI-driven automated medical history–taking system have been presented in previous reports [23,35]. This system converts data entered by patients on tablet terminals into medical terms. First, patients input their age and sex, and then they input their chief concerns as free text. The system then asks approximately 20 questions, one by one, which are optimized based on the previous answers. Finally, physicians can view the entered data as a summarized medical history with the top 10 possible differential diagnoses and their ranks. The diagnostic accuracy, defined as the presence of a final diagnosis in the list of the top 10 possible differential diagnoses, was reported to be 50% based on the data of patients who were unexpectedly admitted within 14 days of the initial outpatient visit [23]. We chose the AI-driven automated medical history–taking system for this study due to its widespread use across Japan, with more than a thousand health care facilities using it. Isabel Pro is a widely used differential diagnostic generator, and its diagnostic accuracy has been validated in several studies [26]. Isabel Pro allows users to input all key findings simultaneously in the form of natural language queries [36]. After entering the queries, Isabel Pro develops the differential diagnoses as a ranked list. The diagnostic accuracy of Isabel Pro was reported to be 89% in a previous systematic review, although the definition varied and heterogeneity was high [26]. We opted for Isabel Pro in this study due to its international recognition as one of the most thoroughly validated systems globally. The AI diagnostic support system for general internal medicine is a diagnostic generator freely available on the internet. This system uses learning-to-rank prediction algorithms with a listwise approach, which is similar to the DDx process of experienced physicians [37]. This system generates possible differential diagnoses by selecting several symptoms or signs from a database that can be searched using a search box. Although the data came from the previous version of the system, the percentage of cases in which the final diagnosis was listed in the top 20 differential diagnoses was reported to be 50% using cases that were difficult to diagnose [34]. We selected the AI diagnostic support system for general internal medicine for this study owing to its accessibility, as it is freely available and supports both English and Japanese languages.

### Outcomes

The primary outcome measure was the prevalence of cases in which the correct diagnosis was included in the DDx list. Two research physicians (RK and MY) independently judged whether the correct diagnosis was included in the DDx list, and conflicts were resolved through discussion. We compared the prevalence of cases in which the correct diagnosis was listed between the index list and the combined DDx lists made from 2 or 3 DDx generators. The combined DDx lists were developed in three patterns:

1. Simply combining differential diagnoses from the index, second, and third lists, excluding the duplicated diagnoses. This means that when the index, second, and third lists did not contain any shared differential diagnoses, the number of differential diagnoses resulted in a total of 20 when combining the index and second or third lists, and the number of differential diagnoses resulted in a total of 30 when combining the 3 lists.
2. Making a new top 10 DDx list by using a proportionally weighted algorithm with a 1/n weighting rule that was used in a previous study [31]. In summary, we weighted each diagnosis in order in each DDx list to downweigh the diagnoses with lower ranks. The weights of each diagnosis among DDx generators were summed to produce the top 10 ranked list of diagnoses.
3. Making new lists with only shared diagnoses among DDx lists from the index, second, and third lists (the minimum number of differential diagnoses could be 0 and the maximum number of differential diagnoses could be 10).

Beyond patterns (1) and (3), we have established diagnostic accuracy as the inclusion of the correct diagnosis within the top 10 differential diagnoses. To our knowledge, there is no validated consensus on defining the diagnostic accuracy of symptom checkers or DDx generators. The AI-driven automated medical history–taking system typically furnishes a list of the top 10 differential diagnoses within standard clinical practice. Therefore, we contend that assessing diagnostic accuracy by identifying the correct diagnosis within the top 10 is a reasonable approach.

### Statistical Analysis

Continuous variables are presented as medians. Categorical variables are presented as numbers and percentages with 95% CIs. The McNemar test was used to assess the improvement in the proportion between the final diagnosis included in the index list and that of the combined DDx lists. In addition to the baseline characteristics of the patients, we treated the data generated by 2 research physicians from the same patient as independent cases. Therefore, the number of cases included in analyses in the case using an additional list was 412 (103 cases × 2 physicians input × 2 DDx generators), and the number of cases included in analyses in the case using 2 additional lists was 206 (103 cases × 2 physicians input). As an exploratory analysis, we also assessed the relationship between the diagnostic accuracy of the AI-driven automated medical history–taking system and the number of shared diagnoses with the other differential diagnostic generators using univariable logistic regression models. In these models, diagnostic accuracy (correct or incorrect) was treated as a binary dependent variable, and the number of shared diagnoses was treated as a continuous independent variable. These analyses were also conducted in the subgroups of common and uncommon diseases, respectively. *P* values below .05 were considered significant. All statistical analyses were conducted using R (version 4.1.0; R Foundation for Statistical Computing).

## Results

### Patient Characteristics

A total of 103 patients were included in this study. Age categories were as follows: 65 years or older: 60 (58%); 50-64 years: 26 (25%); 40-49 years: 9 (9%); 30-39 years: 6 (6%); and 18-29 years: 2 (2%). There were 59 (57%) male patients. General abdominal pain (n=18, 18%) was the most common chief concern, followed by rectal bleeding (n=13, 13%) and shortness of breath (n=12, 12%). Sixty-four diseases were common diseases and 39 were uncommon diseases. The most common category of the final diagnosis was the digestive system (n=36, 35%), followed by the circulatory system (n=17, 17%) and neoplasms (n=15, 15%). The top 10 final diagnoses are shown in Table 1.

**Table 1:** The top 10 final diagnoses (N=103).

Diagnoses	Participants, n (%)
Diverticulosis (diverticular bleeding, diverticulitis)	10 (10)
Colon cancer	7 (7)
Heart failure	7 (7)
Ischemic colitis	6 (6)
Arrhythmia (atrial fibrillation, atrial flutter, sick sinus syndrome, complete atrioventricular block)	6 (6)
Acute appendicitis	4 (4)
Bowel obstruction	4 (4)
Bacterial pneumonia	4 (4)
Acute pyelonephritis	4 (4)
Diabetes mellitus	4 (4)

### DDx Lists

The median number of shared diagnoses between the DDx lists of the AI-driven automated medical history–taking system and the other DDx generators was 2 (range 0-6), and the median number of shared diagnoses in all 3 DDx lists was 1 (range 0-4).

### Outcomes

The proportion of cases in which the final diagnosis was listed in the DDx list of the AI-driven automated medical history–taking system was 47/103 (46%, 95% CI 36%-56%). The average proportion of the cases in which a final diagnosis was listed in the DDx list of Isabel Pro and the AI diagnostic support system for general internal medicine was 84/206 (41%, 95% CI 34%-48%) and 55/206 (27%, 95% CI 21%-33%), respectively.

**Figure 1:**
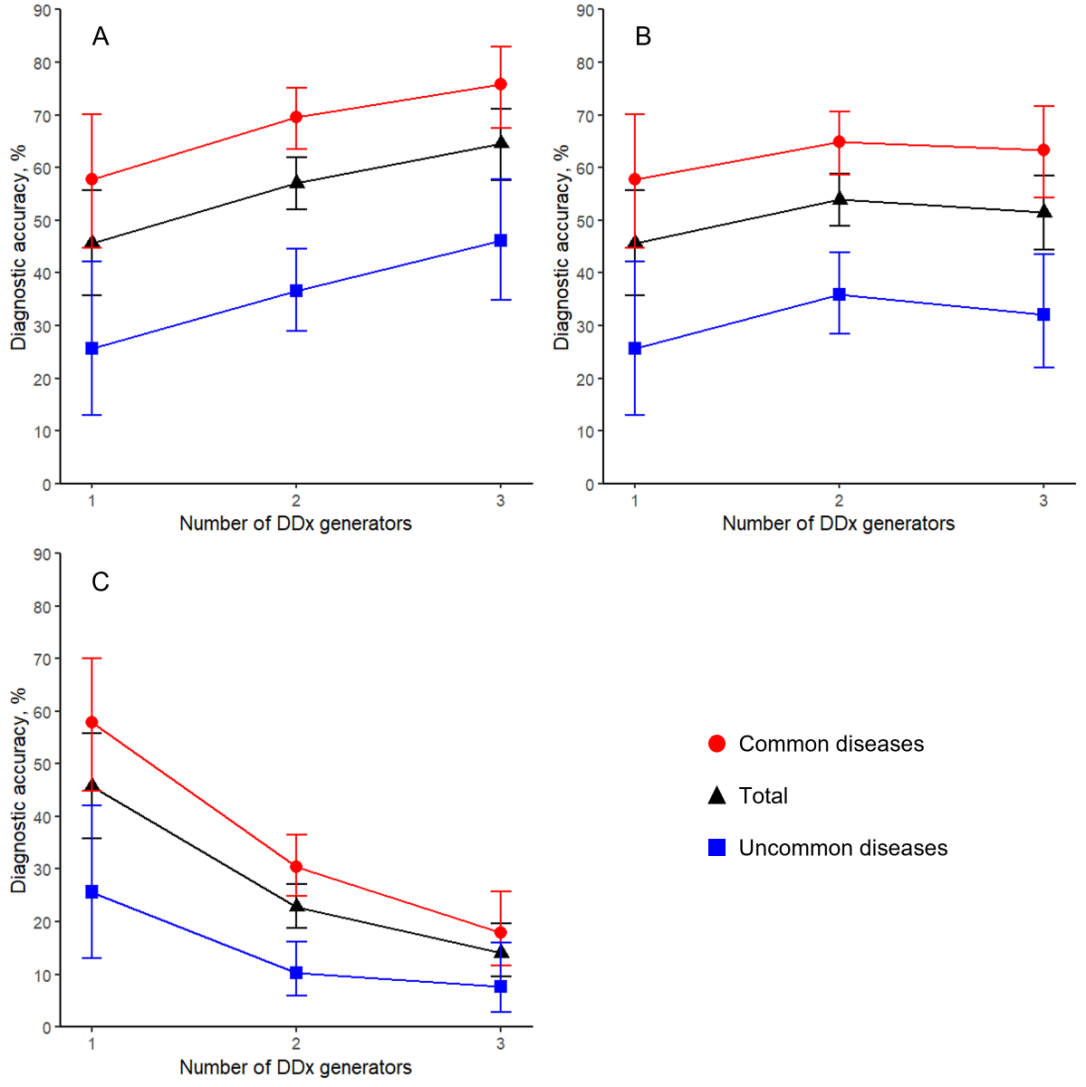
The likelihood of the correct diagnosis being present in the single and combined DDx lists. The y-axis represents the likelihood of the correct diagnosis being present in the DDx lists, and the x-axis represents the number of DDx generators used for combined DDx lists. Error bars are 95% CIs. (A) Combined DDx lists are made by simply adding differential diagnoses from the DDx generators used. (B) Combined top 10 DDx lists made by using the 1/n weighting rule (eg, the first diagnosis of each list has a weight of 1, the second is 1/2, and so on). (C) Combined DDx lists made by only shared differential diagnoses among DDx generators. Diagnostic accuracies are shown for the total group of patients (red circles) and subgroups with common (black triangles) and uncommon (blue squares) diseases. DDx: differential diagnosis.

The proportion of the final diagnosis included in the combined DDx list of the AI-driven automated medical history–taking system and the other DDx generator (ie, the combination of 2 DDx generators) was 235/412 (57%, 95% CI 52%-62%, McNemar test, *P*<.001) in the simply added list (Figure 1), 222/412 (54%, 95% CI 49%-59%, McNemar test, *P*<.001) in the collective list with 1/n weighting rule (Figure 1), and 94/412 (23%, 95% CI 19%-27%, McNemar test, *P*<.001) in the shared list (Figure 1). The proportion of the final diagnosis included in the combined DDx list of all 3 DDx lists was 133/206 (65%, 95% CI 58%-71%, McNemar test, *P*<.001) in the simply added list (Figure 1), 106/206 (52%, 95% CI 44%-59%, McNemar test, *P*=.05) in the collective list with 1/n weighting rule (Figure 1), and 29/206 (14%, 95% CI 10%-20%, McNemar test, *P*<.001) in the shared list (Figure 1). Figure 1 and Table 2 also present data stratified according to disease commonality. These results indicate that trends observed among patients with both common and uncommon diseases parallel the overall trends identified within the total patient cohort.

**Figure 2:**
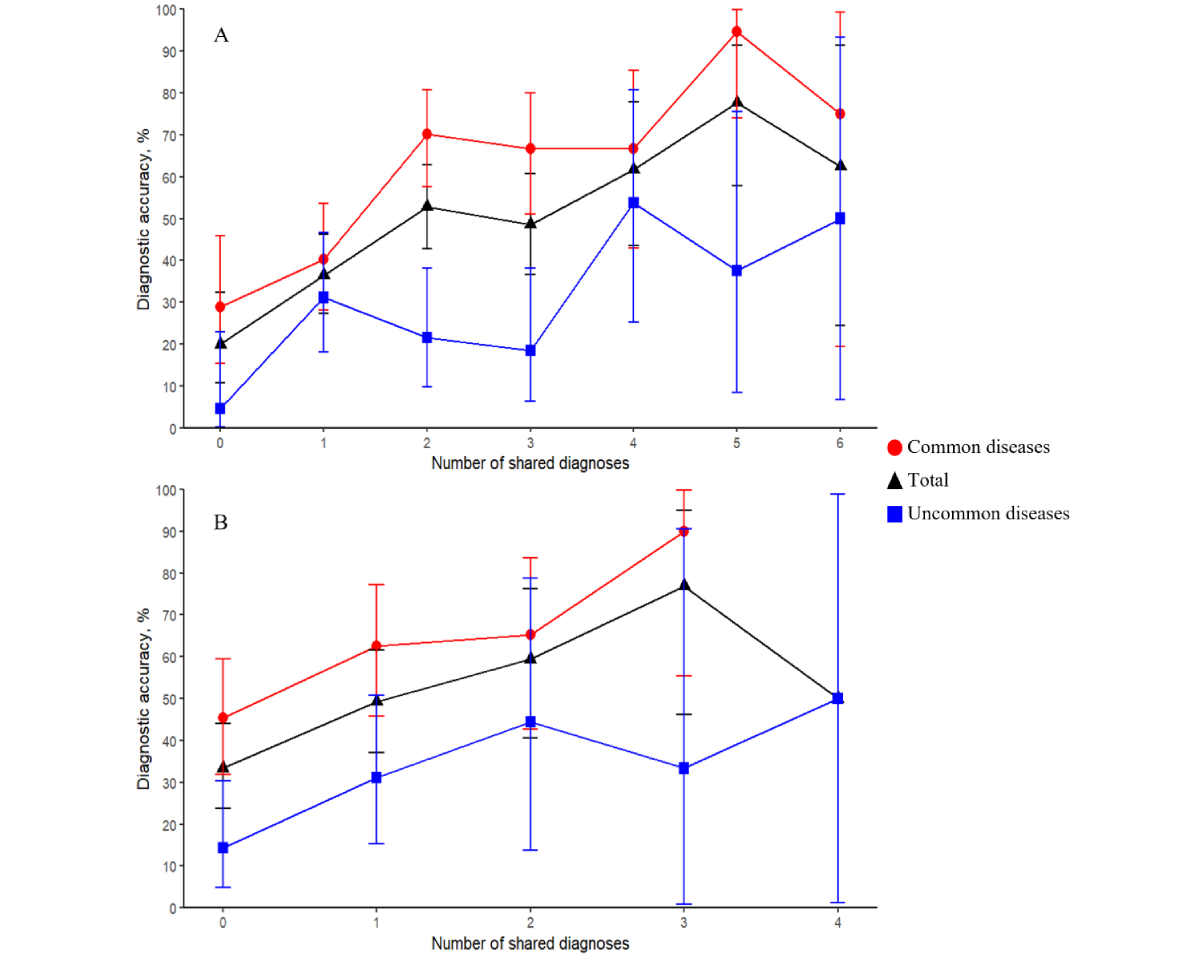
Diagnostic accuracy of the top 10 differential diagnosis (DDx) lists developed by an artificial intelligence–driven automated medical history–taking system based on the number of shared differential diagnoses with the other top 10 DDx lists developed by other DDx generators. The y-axis indicates accuracy (correct diagnosis included in the DDx lists), and the x-axis indicates the number of shared differential diagnoses between the top 10 DDx lists developed by the artificial intelligence–driven automated medical history–taking system and the DDx lists developed by other DDx generators. Error bars are 95% CIs. (A) In the case of using 1 additional DDx generator. (B) In the case of using 2 additional DDx generators. Diagnostic accuracies are shown for the total group of patients (red circles) and subgroups with common (black triangles) and uncommon (blue squares) diseases.

In the logistic regression models, the number of shared differential diagnoses with 1 additional diagnostic generator was significantly associated with the diagnostic accuracy of the DDx list of the AI-driven automated medical history–taking system (from 20% in the cases with no shared DDx to 78% in the cases with 5 shared differential diagnoses; odds ratio 1.48 for each one shared differential diagnoses increase, 95% CI 1.29-1.72; *P*<.001; Figure 2) and the number of shared differential diagnoses with 2 additional diagnostic generators was also significantly associated with the diagnostic accuracy of the DDx list of the AI-driven automated medical history–taking system (from 33% in the cases with no shared differential diagnoses to 77% in the cases with 3 shared differential diagnoses; odds ratio 1.70 for each one shared differential diagnoses increase, 95% CI 1.26-2.35; *P*<.001; Figure 2). These trends were also observed when the data were stratified by disease commonality (Figure 2 and Table 3).

**Table 2:** Diagnostic accuracy of combined differential diagnosis lists.

			Correct/total, n (%)				
			Total (N=103)		Common diseases (n=64)		Uncommon diseases (n=39)
**List of 1 system**							
	Index list	47/103 (46)		37/64 (58)		10/39 (26)	
**Combined lists of 2 systems**							
	Simply added list	235/412 (57)		178/256 (70)		57/156 (37)	
	Collective list with 1/n weighting rule	222/412 (54)		166/256 (65)		56/156 (36)	
	Shared list	94/412 (23)		78/256 (31)		16/156 (10)	
**Combined lists of 3 systems**							
	Simply added list	133/206 (65)		97/128 (76)		36/78 (46)	
	Collective list with 1/n weighting rule	106/206 (52)		81/128 (63)		25/78 (32)	
	Shared list	29/206 (14)		23/128 (18)		6/78 (8)	

**Table 3:** Odds ratios of the number of shared differential diagnoses between the artificial intelligence (AI)–driven automated medical history–taking system and other differential diagnosis (DDx) generators for the diagnostic accuracy of the DDx list of the AI-driven automated medical history–taking system.

		OR^a,b^ (95% CI)	*P* value
**With 1 additional system**			
	Total	1.48 (1.29-1.72)	<.001
	Common diseases	1.70 (1.40-2.10)	<.001
	Uncommon diseases	1.32 (1.04-1.68)	.02
**With 2 additional systems**			
	Total	1.70 (1.26-2.35)	<.001
	Common diseases	1.77 (1.19-2.72)	.01
	Uncommon diseases	1.68 (1.01-2.89)	.049

^a^OR: odds ratio.

^b^For each one shared differential diagnoses increase.

## Discussion

### Principal Results

This study showed that simply adding DDx lists from other DDx generators to the DDx list of AI-driven automated medical history–taking systems increases the likelihood of correct diagnoses being present in the DDx list. In addition, this study demonstrated that the number of shared differential diagnoses with additional DDx generators was associated with the diagnostic accuracy of the DDx list of AI-driven automated medical history–taking systems.

### Comparison With Prior Work

This result was consistent with that of a previous study that showed that using DDx support early in the diagnostic process increased the number of differential diagnoses and the likelihood of the correct diagnosis being present in the DDx list of physicians and medical students [16]. Based on the results of this study, an approximately 10% increase in the likelihood of a correct diagnosis being present in the DDx list was achieved by simply adding the top 10 differential diagnoses from 1 additional DDx generator. This result is important because a previous study reported that providing a diagnosis list without a correct diagnosis did not improve and might have slightly reduced diagnostic accuracy [38]. While this approach can increase the likelihood of a correct diagnosis being present in the differential diagnoses, there is a disadvantage in that it can increase the number of differential diagnoses. However, notably, clinicians demonstrating consistent accuracy tend to incorporate a larger number of items in their diagnostic lists compared to their less accurate counterparts [39]. Furthermore, a separate study indicated that diagnostic checklists encompassing more than 30 differential diagnoses enhanced diagnostic accuracy among medical students [40]. In fact, a significant number of symptom checkers offer more than 10 differential diagnoses [41]. Thus, certain clinicians may be amenable to accepting an addition of 10 to 20 differential diagnoses if such a change can lead to an improvement of 10% to 20% in diagnostic accuracy. Conversely, other clinicians may require more cost-effective methods. Therefore, other approaches for the combinational use of DDx generators that can increase accuracy without increasing the number of differential diagnoses are favored. Based on this background, we assessed 2 other approaches: the proportionally weighted algorithm with a 1/n weighting rule and selecting only shared differential diagnoses to make lists of 10 or fewer differential diagnoses in this study. The results showed that selecting only shared differential diagnoses is not recommended. Regarding the approach that used a proportionally weighted algorithm with a 1/n weighting rule, merging the index and another DDx list significantly improved the diagnostic accuracy (by approximately 8%) without increasing the number of differential diagnoses, whereas merging all 3 DDx lists did not improve the diagnostic accuracy. This study’s results suggest that this approach may function well when using 1 additional DDx generator but not when using 2 or more generators. This result is consistent with the results of previous studies that assessed the collective intelligence of physicians and medical students, which showed that the range of improvement in diagnostic accuracy of the collective DDx list made by the 1/n weighting rule or the mean of each individual output was largest between individuals and groups of 2 persons [31,42].

The results of this study also demonstrate the potential usefulness of additional DDx generators as indicators of the trustworthiness of the lists of DDx generators. In this study, there was a trend that as there were more shared diagnoses with the other DDx generators, the diagnostic accuracy of the DDx list of AI-driven automated medical history–taking systems was higher. As an example of applying the results of this study to clinical practice, a strategy that does not trust the quality of medical history developed by the AI-driven automated medical history–taking system when the number of shared differential diagnoses is zero or low may be efficient in preventing diagnostic errors because the DDx list of AI-driven automated medical history–taking systems depends on the medical history of the system. However, because these results were obtained from exploratory analyses, further validation studies are required.

### Limitations

This study had several limitations. First, although this study used data from an AI-driven automated medical history–taking system obtained from real patients, it is unknown whether the combined use of other differential diagnostic generators can improve the diagnostic accuracy or quality of the diagnostic process for physicians. Second, because this study used only 2 specific DDx generators, it is unknown how many and what kind of DDx generators should be used to maximize diagnostic accuracy. Third, the patients were included from only 1 hospital in Japan, and gastrointestinal diseases, cardiovascular diseases, and neoplasms were common diagnostic categories. Therefore, the results of this study may not be generalizable to other populations.

### Conclusions

Simply combining the DDx lists developed by other DDx generators with the DDx lists of AI-driven automated medical history–taking systems may improve the likelihood of a correct diagnosis in the new DDx list. However, future studies are warranted to determine the optimal number and strategies for the combined use of DDx generators to maximize diagnostic accuracy.

## Abbreviations

AI: artificial intelligence
DDx: differential diagnosis

## References

[1] Fernholm R, Härenstam KP, Wachtler C, Nilsson GH, Holzmann MJ, Carlsson AC (2019). Diagnostic errors reported in primary healthcare and emergency departments: a retrospective and descriptive cohort study of 4830 reported cases of preventable harm in Sweden. Eur J Gen Pract.

[2] Singh H, Schiff GD, Graber ML, Onakpoya I, Thompson MJ (2017). The global burden of diagnostic errors in primary care. BMJ Qual Saf.

[3] Avery AJ, Sheehan C, Bell B, Armstrong S, Ashcroft DM, Boyd MJ, Chuter A, Cooper A, Donnelly A, Edwards A, Evans HP, Hellard S, Lymn J, Mehta R, Rodgers S, Sheikh A, Smith P, Williams H, Campbell SM, Carson-Stevens A (2021). Incidence, nature and causes of avoidable significant harm in primary care in England: retrospective case note review. BMJ Qual Saf.

[4] Cheraghi-Sohi S, Holland F, Singh H, Danczak A, Esmail A, Morris RL, Small N, Williams R, de Wet C, Campbell SM, Reeves D (2021). Incidence, origins and avoidable harm of missed opportunities in diagnosis: longitudinal patient record review in 21 English general practices. BMJ Qual Saf.

[5] Singh H, Meyer AND, Thomas EJ (2014). The frequency of diagnostic errors in outpatient care: estimations from three large observational studies involving US adult populations. BMJ Qual Saf.

[6] Khoo EM, Lee WK, Sararaks S, Abdul Samad A, Liew SM, Cheong AT, Ibrahim MY, Su SHC, Mohd Hanafiah AN, Maskon K, Ismail R, Hamid MA (2012). Medical errors in primary care clinics–a cross sectional study. BMC Fam Pract.

[7] Aoki T, Watanuki S (2020). Multimorbidity and patient-reported diagnostic errors in the primary care setting: multicentre cross-sectional study in Japan. BMJ Open.

[8] Harada Y, Otaka Y, Katsukura S, Shimizu T (2023). Effect of contextual factors on the prevalence of diagnostic errors among patients managed by physicians of the same specialty: a single-centre retrospective observational study. BMJ Qual Saf.

[9] Singh H, Thomas EJ, Khan MM, Petersen LA (2007). Identifying diagnostic errors in primary care using an electronic screening algorithm. Arch Intern Med.

[10] Singh H, Giardina TD, Meyer AND, Forjuoh SN, Reis MD, Thomas EJ (2013). Types and origins of diagnostic errors in primary care settings. JAMA Intern Med.

[11] Harada T, Miyagami T, Watari T, Hiyoshi T, Kunitomo K, Tsuji T, Shimizu T (2021). Analysis of diagnostic error cases among Japanese residents using diagnosis error evaluation and research taxonomy. J Gen Fam Med.

[12] Schiff GD, Volodarskaya M, Ruan E, Lim A, Wright A, Singh H, Reyes Nieva H (2022). Characteristics of disease-specific and generic diagnostic pitfalls: a qualitative study. JAMA Netw Open.

[13] Schiff GD, Hasan O, Kim S, Abrams R, Cosby K, Lambert BL, Elstein AS, Hasler S, Kabongo ML, Krosnjar N, Odwazny R, Wisniewski MF, McNutt RA (2009). Diagnostic error in medicine: analysis of 583 physician-reported errors. Arch Intern Med.

[14] Kostopoulou O, Rosen A, Round T, Wright E, Douiri A, Delaney B (2015). Early diagnostic suggestions improve accuracy of GPs: a randomised controlled trial using computer-simulated patients. Br J Gen Pract.

[15] Kostopoulou O, Lionis C, Angelaki A, Ayis S, Durbaba S, Delaney BC (2015). Early diagnostic suggestions improve accuracy of family physicians: a randomized controlled trial in Greece. Fam Pract.

[16] Sibbald M, Monteiro S, Sherbino J, LoGiudice A, Friedman C, Norman G (2022). Should electronic differential diagnosis support be used early or late in the diagnostic process? A multicentre experimental study of Isabel. BMJ Qual Saf.

[17] Zakim D (2016). Development and significance of automated history-taking software for clinical medicine, clinical research and basic medical science. J Intern Med.

[18] Berdahl CT, Henreid AJ, Pevnick JM, Zheng K, Nuckols TK (2022). Digital tools designed to obtain the history of present illness from patients: scoping review. J Med Internet Res.

[19] Zakim D, Brandberg H, El Amrani S, Hultgren A, Stathakarou N, Nifakos S, Kahan T, Spaak J, Koch S, Sundberg CJ (2021). Computerized history-taking improves data quality for clinical decision-making–comparison of EHR and computer-acquired history data in patients with chest pain. PLoS One.

[20] Almario CV, Chey W, Kaung A, Whitman C, Fuller G, Reid M, Nguyen K, Bolus R, Dennis B, Encarnacion R, Martinez B, Talley J, Modi R, Agarwal N, Lee A, Kubomoto S, Sharma G, Bolus S, Chang L, Spiegel BMR (2015). Computer-generated vs. physician-documented history of present illness (HPI): results of a blinded comparison. Am J Gastroenterol.

[21] Matsuoka A, Miike T, Yamazaki H, Higuchi M, Komaki M, Shinada K, Nakayama K, Sakurai R, Asahi M, Yoshitake K, Narumi S, Koba M, Sugioka T, Sakamoto Y (2022). Usefulness of a medical interview support application for residents: a pilot study. PLoS One.

[22] Schwitzguebel AJP, Jeckelmann C, Gavinio R, Levallois C, Benaïm C, Spechbach H (2019). Differential diagnosis assessment in ambulatory care with an automated medical history-taking device: pilot randomized controlled trial. JMIR Med Inform.

[23] Kawamura R, Harada Y, Sugimoto S, Nagase Y, Katsukura S, Shimizu T (2022). Incidence of diagnostic errors among unexpectedly hospitalized patients using an automated medical history-taking system with a differential diagnosis generator: retrospective observational study. JMIR Med Inform.

[24] Wallace W, Chan C, Chidambaram S, Hanna L, Iqbal FM, Acharya A, Normahani P, Ashrafian H, Markar SR, Sounderajah V, Darzi A (2022). The diagnostic and triage accuracy of digital and online symptom checker tools: a systematic review. NPJ Digit Med.

[25] Chambers D, Cantrell AJ, Johnson M, Preston L, Baxter SK, Booth A, Turner J (2019). Digital and online symptom checkers and health assessment/triage services for urgent health problems: systematic review. BMJ Open.

[26] Riches N, Panagioti M, Alam R, Cheraghi-Sohi S, Campbell S, Esmail A, Bower P (2016). The effectiveness of electronic differential diagnoses (DDX) generators: a systematic review and meta-analysis. PLoS One.

[27] Harada Y, Katsukura S, Kawamura R, Shimizu T (2021). Efficacy of artificial-intelligence-driven differential-diagnosis list on the diagnostic accuracy of physicians: an open-label randomized controlled study. Int J Environ Res Public Health.

[28] Harada Y, Katsukura S, Kawamura R, Shimizu T (2021). Effects of a differential diagnosis list of artificial intelligence on differential diagnoses by physicians: an exploratory analysis of data from a randomized controlled study. Int J Environ Res Public Health.

[29] Kämmer JE, Hautz WE, Herzog SM, Kunina-Habenicht O, Kurvers RHJM (2017). The potential of collective intelligence in emergency medicine: pooling medical students' independent decisions improves diagnostic performance. Med Decis Making.

[30] Hautz WE, Kämmer JE, Schauber SK, Spies CD, Gaissmaier W (2015). Diagnostic performance by medical students working individually or in teams. JAMA.

[31] Barnett ML, Boddupalli D, Nundy S, Bates DW (2019). Comparative accuracy of diagnosis by collective intelligence of multiple physicians vs individual physicians. JAMA Netw Open.

[32] Radcliffe K, Lyson HC, Barr-Walker J, Sarkar U (2019). Collective intelligence in medical decision-making: a systematic scoping review. BMC Med Inform Decis Mak.

[33] Khoong EC, Nouri SS, Tuot DS, Nundy S, Fontil V, Sarkar U (2022). Comparison of diagnostic recommendations from individual physicians versus the collective intelligence of multiple physicians in ambulatory cases referred for specialist consultation. Med Decis Making.

[34] Torigoe K, Tokuda Y (2016). Potential usefulness of diagnostic reminder as web-based clinical decision support system. J Health Sci.

[35] Harada Y, Shimizu T (2020). Impact of a commercial artificial intelligence-driven patient self-assessment solution on waiting times at general internal medicine outpatient departments: retrospective study. JMIR Med Inform.

[36] Bond WF, Schwartz LM, Weaver KR, Levick D, Giuliano M, Graber ML (2012). Differential diagnosis generators: an evaluation of currently available computer programs. J Gen Intern Med.

[37] Miyachi Y, Ishii O, Torigoe K (2023). Design, implementation, and evaluation of the computer-aided clinical decision support system based on learning-to-rank: collaboration between physicians and machine learning in the differential diagnosis process. BMC Med Inform Decis Mak.

[38] Kämmer JE, Schauber SK, Hautz SC, Stroben F, Hautz WE (2021). Differential diagnosis checklists reduce diagnostic error differentially: a randomised experiment. Med Educ.

[39] Krupat E, Wormwood J, Schwartzstein RM, Richards JB (2017). Avoiding premature closure and reaching diagnostic accuracy: some key predictive factors. Med Educ.

[40] Shimizu T, Matsumoto K, Tokuda Y (2013). Effects of the use of differential diagnosis checklist and general de-biasing checklist on diagnostic performance in comparison to intuitive diagnosis. Med Teach.

[41] Hill MG, Sim M, Mills B (2020). The quality of diagnosis and triage advice provided by free online symptom checkers and apps in Australia. Med J Aust.

[42] Stehouwer NR, Torrey KW, Dell MS (2023). Collective intelligence improves probabilistic diagnostic assessments. Diagnosis (Berl).

